# Ascending cholangitis presenting with *Lactococcus lactis cremoris *bacteraemia: a case report

**DOI:** 10.1186/1752-1947-3-3

**Published:** 2009-01-06

**Authors:** Jane Davies, Michael David Burkitt, Alastair Watson

**Affiliations:** 1 Tropical and Infectious Diseases Unit, Royal Liverpool and Broadgreen University Hospitals Trust, Prescot Street, Liverpool, L7 8XP, UK; 2 The Henry Wellcome Laboratories, Unit of Gastroenterology, School of Clinical Science, 1st Floor Nuffield Building, Ashton Street, The University of Liverpool, Liverpool, L69 3GE, UK

## Abstract

**Introduction:**

A case of *Lactococcus lactis cremoris *causing cholangitis is described. This Gram-positive organism is not routinely considered to be pathogenic in immunocompetent individuals. To our knowledge, this is the thirteenth report of invasive infection and the first of cholangitis to be reported in association with this organism.

**Case presentation:**

A 72-year-old patient presented with Charcot's triad and was demonstrated to have cholangitis with *Lactococcus lactis cremoris *bacteraemia. Biliary drainage was achieved through endoscopic retrograde cholangiography. Antibiotic therapy with multiple agents was necessary.

**Conclusion:**

This report provides corroboration of evidence that *Lactococcus lactis cremoris *is a potential pathogen in immunocompetent adults. There remains a debate about the most appropriate empirical antibiotic therapy in this condition. In the light of this case, it is important to keep an open mind to potential pathogens.

## Introduction

*Lactococcus lactis cremoris *is commonly considered to be a non-pathogenic organism in humans. It is recognized as a commensal organism of mucocutaneous surfaces, however, over the past 50 years, there have been a number of case reports [1-11] demonstrating the potential for this organism to cause infection. We report the first case of cholangitis associated with septicaemia caused by *Lactococcus lactis cremoris*.

## Case presentation

A 72-year-old lady, normally fit and well, presented with a 5-day history of jaundice and abdominal pain. She was nauseated and had dark urine. On initial assessment, she was deeply icteric and her temperature was 38.2°C but she was haemodynamically stable. Systemic examination did not reveal any other abnormalities, specifically there were no stigmata of chronic liver disease. No organs or lymph nodes were palpable and the abdomen was soft and non-tender.

Biochemical analyses demonstrated a leukocytosis and neutrophilia; haemoglobin (Hb) 11.9 g/dL, white blood cell count (WCC) 13.9 × 10^9^/L, neutrophils 11.4 × 10^9^/L. An acute phase response was evident with C-reactive protein (CRP) 131 mg/L. A mixed cholestatic and hepatic picture of hepatic enzymes with alkaline phosphatase (ALP) 340 U/L, alanine aminotransferase (ALT) 240 U/L and gamma-glutamyl-transferase (γGT) 381 U/L was demonstrated; total bilirubin was 351 μmol/L. Hepatic synthetic function was preserved with albumin 30 g/L and prothrombin time (PT) of 13.8 seconds. A clinical diagnosis of cholangitis was made on the basis of Charcot's triad (abdominal pain, fever and jaundice), and empirical antibiotic therapy (oral ciprofloxacin 500 mg bd) was commenced.

An ultrasound of the biliary tree was performed demonstrating dilatation of the common bile duct to 1.5 cm with visualization of at least one stone in the lumen of the duct. Intrahepatic duct dilatation was also noted. Blood cultures confirmed a *Lactococcus lactis cremoris *septicaemia. The organism was sensitive to tazobactam/piperacillin and co-amoxiclav. In light of these results, antibiotic therapy was changed to intravenous tazobactam/piperacillin 4.5 g tds.

The patient proceeded to endoscopic retrograde cholangiopancreatogram (ERCP) where an impacted common bile duct stone was identified. Unfortunately, this was not amenable to endoscopic removal despite sphincterotomy; however two biliary stents were inserted with good drainage.

The patient recovered rapidly with resolution of her symptoms and signs and was discharged home 48 hours post-ERCP. Treatment was completed with 2 weeks of oral co-amoxiclav 625 mg tds.

## Discussion

The Tokyo Consensus guidelines of 2007 have now established definitive diagnostic criteria and severity assessment of cholangitis [12]. The diagnosis of cholangitis is made either by the presence of Charcot's triad or by the presence of two of these features backed up by abnormal liver function tests, raised inflammatory markers and imaging demonstrating a dilated biliary tree. Severity is assessed by the presence or absence of organ failure once a diagnosis has been made and response to initial therapy. As our patient had no signs of organ failure but failed to respond to the primary treatment, she constitutes cholangitis of moderate severity.

Empirical antibiotic therapy for cholangitis is targeted towards gut organisms, particularly Gram-negative organisms. Commonly (including in our unit), ciprofloxacin is considered to be an appropriate empirical therapy. This is backed up by reports of an 85% clinical cure rate in trials [13]. The Tokyo Consensus group [13] failed to recommend a single specific empirical treatment, therefore local antibiotic guidelines will continue to direct empirical therapy. In the presence of positive microbiological investigations, there is a clear consensus that agents should be changed for more appropriate treatment according to sensitivity.

Biliary drainage reduces mortality and speeds recovery from cholangitis and is therefore a vital part of management [14]. The Tokyo guidelines recognize that this must be done in an emergency setting for patients with severe cholangitis and as promptly as practical in other patients. Endoscopic drainage is the preferred modality [15].

*Lactococcus lactis cremoris *is a Gram-positive coccus, formerly classified as *Streptococcus cremoris *but now recognized as a member of the genus *Lactococcus *[3]. This species is commonly regarded as non-pathogenic in immunocompetent adults, however we report the thirteenth case to our knowledge of this pathogen causing clinically significant infection. Previously, four cases of bacterial endocarditis [4,6,9,11], one of septicaemia [7], two liver abscesses [3,5] and one each of necrotizing pneumonitis [10], septic arthritis [8], deep neck infection [2], cerebellar abscess [4] and canaliculitis [1] have been reported. Of these, it appears that nine were immunocompetent patients. All bar one of the case reports were in adults (Table 1).

**Table 1:** Previously reported cases of *Lactococcus lactis cremoris *associated infections

Year	Age	Sex	Site of infection	Exposure to unpasteurized milk products	Treatment	Outcome	Immune status
2006 [1]	80	F	Canaliculitis	None	Oral ampicillin and topical chloramphenicol	Complete resolution	Normal
2005 [2]	68	M	Deep neck infection	Cow breeder and consumed unpasteurized milk	Ceftriaxone and metronidazole for 6 weeks	Resolution on discharge	Previous malignancy
2004 [3]	79	F	Liver abscess	None	Percutaneous drainage, Imipenem Cilastatin for 5 weeks	Complete resolution	Normal
2002 [4]	45	F	Cerebellar abscess	Not commented	Ceftriaxone 8 weeks, gentamicin 2 weeks, Metronidazole	No residual deficit and no recurrence at 9 months	Normal
2002 [3]	67	M	Endocarditis	History of drinking unpasteurized milk	Co-amoxiclav and gentamicin 15 days	Well 6 months post discharge	Normal
					Penicillin for 6 weeks		
2000 [5]	14	F	Liver abscess	None	Percutaneous drainage	Discharged from hospital on day 48	Normal
					Cefotiam, Amikacin and Clindamycin for 8 days		
					Panipenem for 8 days		
					Piperacillin 15 days and amikacin 10 days		
1996 [6]	56	M	Endocarditis	None	Penicillin G for 12 days and Clarithromycin for 18 days	Well 18 months post discharge	Normal
1995 [7]	69	M	Septicaemia	Yoghurt ingested	Cefotaxime and Amikacin	No comment	Chronic lymphocytic leukaemia
1993 [8]	57	F	Septic arthritis	Unpasteurized milk	Penicillin for 6 weeks	Deformity 8 months post discharge, but no ongoing infection	Normal
1990 [9]	65	F	Endocarditis	Not commented	Benzylpenicillin and gentamicin	No ongoing infection	Normal
1990 [10]	24	M	Necrotizing pneumonitis and empyema	Unpasteurized milk and cheese eaten	Thoracocentesis (*3) Penicillin and clindamycin 15 days	Well 1 month post discharge	HIV positive
1955 [11]	21	M	Endocarditis	Sour cream known to contain *S. Lactis*	Penicillin and Dihydrostreptomycin for 22 days	Well 4 months post discharge	Normal

*Lactococcus lactis cremoris *is a recognized skin commensal of cattle and is also used in the dairy industry for milk fermentation. It may therefore be present in unpasteurized dairy products. Of the previously reported cases, six have been associated with a clear history of exposure to unpasteurized dairy products; in one of these cases, the organism was isolated from the milk product (Table 1). Our patient is not aware of having had any such exposure.

## Conclusion

This report provides corroboration of evidence that *Lactococcus lactis cremoris *is a potential pathogen in immunocompetent adults. *Lactococcus lactis cremoris *has now been reported as a pathogen in many different systems, both acutely and subacutely. This may well represent an underreporting of the true incidence of invasive infection related to this organism.

Diagnosis and assessment of the clinical severity of cholangitis are now the subject of consensus guidelines. These guidelines also extend to the appropriate timing and method of biliary drainage. However, there remains a debate about the most appropriate empirical antibiotic therapy in this condition. In the light of this case, it is important to consider other potential pathogens causing ascending cholangitis.

## Abbreviations

Hb: haemoglobin; WCC: white cell count; CRP: C-reactive protein; ALT: alanine aminotransferase; ALP: alkaline phosphatase; γGT: gamma-glutamyl-transferase; PT: prothrombin time; bd: twice daily; tds: three times daily; ERCP: endoscopic retrograde cholangiopancreatogram

## Consent

Written informed consent was obtained from the patient for publication of this case report and any accompanying images. A copy of the written consent is available for review by the Editor-in-Chief of this journal.

## Competing interests

The authors declare that they have no competing interests.

## Authors' contributions

JD and MDB were involved in patient care, carried out the review of literature and were jointly responsible for drafting and revising the manuscript. AJMW has provided editorial and clinical supervision.
